# Yellow Fever, Considered in Its Historical, Pathological, Etiological, and Therapeutical Relations, Including a Sketch of the Disease as It Has Occurred in Philadelphia

**Published:** 1855-10

**Authors:** 


Art. IV.—*Yellow Fever, considered in its Historical, Pathological, Etiological, and Therapeutical Relations, including a sketch of the Disease as it has occurred in Philadelphia, from* 1699 *to* 1854, *with an examination of the connections between it and the fevers known under the same name in other parts of Temperate, as well as in Tropical, Regions.* By R. La Roche, M. D., Member of the American Philosophical Society; of the American Medical Association; Fellow of the College of Physicians of Philadelphia; Corresponding Member of the Imperial Academy of Medicine, and Foreign Associate of the Medical Society of Emulation, of Paris; of the Academies of Sciences of Turin, Copenhagen, Stockholm, Nancy, and New Orleans, of the Medical Societies of Naples, Marseilles, Lyons, etc. Philadelphia: Blanchard & Lea; 1855.

Dr. La Roche is known to have been for many years engaged in the preparation of a work on yellow fever. It has, at last, appeared, and never could it have made its advent on an occasion more opportune that the present. Yellow fever has within a short period become invested with a new and pervading interest. After a long absence from cities where it once prevailed, it has recently appeared again, and its diffusion over our country has been wider, within a few years, than was ever known before. It has made its appearance in localities where it never existed, and invaded districts where it was thought it could never reach. Its mortality has been very great. This elaborate treatise of Dr. La Roche will, therefore, be hailed by the profession as a most seasonable contribution to our literature on the subject. His work, though treating more especially of the yellow fever in Philadelphia, aims at a more enlarged and important character—at presenting an *expose* of the whole subject, its symptomatology, anatomical characters, pathology, treatment, etiology, and the laws by which it is governed. The task in which the laborious author has been so long engaged has been nobly performed. The result of his pains-taking efforts is a work which reflects credit upon his learning, judgment and taste, and does honor to our country. It is a great work—great for its research, for its historical details, its statistics, its learning, and the spirit of philosophy with which it is instinct. In point of size it also belongs to the class of the great; it is comprised in two large octavo volumes of more than fourteen hundred pages. The reader must be curious about yellow fever who cannot find here every point discussed in which he can feel any interest.

Dr. La Roche prefaces his treatise with an account of the medical topography of Philadelphia, together with a history of yellow fever in that city. We pass over all that he says on the former subject, to give some extracts from his interesting history.

Yellow fever first appeared in Philadelphia in 1699, when scarcely a few hundred families from the mother country had clustered together there, the first settlement having been made seventeen years before. The mortality was proportionally as great as it has ever been in any subsequent visitation of the fever. No professional account of this earliest epidemic has been given. Dr. La Roche gleans a meagre, but instructive, history of it from the journals of the few travelers who visited the place, the manuscript correspondence of one or two of its citizens, and the pages of the early historians of the colonies. It broke out about the middle of June, in a very hot season, and was regarded by the citizens as similar to the fever which scourged Barbadoes and other tropical countries. Mr. Norris, the great-grandfather of the present distinguished Dr. George W. Norris, of Philadelphia, wrote to a friend that the summer, about harvest time had been the hottest that he had ever felt, and added, that he "really thought that several died in the field with the violence of the heat." The number of deaths reported by the grave digger was 220 out of a population of 3,800, or about 1 in 17 of the inhabitants, a mortality that must have been frightful to the colonists.

"Well can we understand the dismay which this wide-spreading mortality created among the peaceful inhabitants of the rising city, as strongly depicted by Story. "Great," he says, "was the majesty and hand of the Lord; great was the fear that fell upon all flesh; I saw no lofty, airy countenance, nor heard any vain jesting to move men to laughter; nor witty repartee to raise mirth; nor extravagant feasting to excite the lusts and desires of the flesh above measure; but every face gathered paleness, and many hearts were humbled and countenances fallen and sunk, as such that waited every moment to be summoned to the bar, and numbered to the grave."

From 1699 to the year 1741 we hear nothing of yellow fever in Philadelphia, during which period it visited many southern ports in the Atlantic and five times prevailed at Charleston. Dr. Bard states that in the year 1802, a malignant fever broke out in New York, which, he says, was but little inferior to the plague, and which was probably yellow fever. It was reported to have been imported from "St. Thomas in a single bale of cotton."

Though Philadelphia maintained as free intercourse as before with the West Indies and the neighboring cities, she remained unscathed during the long period above mentioned, but in 1741, while prevailing in Jamaica, and other West India islands, the fever broke out with violence, and continued until checked by the advent of cold weather. The following extract of a letter written in 1741 by Mr. Peters, quoted by Dr. La Roche, will be read with interest, as containing valuable facts and as setting forth the views of the time in reference to the introduction of the fever.

"At the time of your brother's departure from this city, it was afflicted with a most malignant distemper imported in the spring by ships from Ireland. At first it crept along the wharves, affecting strangers, common sailors, and the low and poor part of the people, and was easily managed by the physicians; but, as the dog-days advanced, having a long and dry season without gusts, the sickness increased and attacked the better sort of the inhabitants, of whom a good many died, though not in such numbers as is reported. About the 17th August, the physicians opened the body of a man who died in forty-eight hours from the first sensible attack, and found his bowels and stomach quite mortified. From the 17th August to 17th September, the distemper continued very violent, and baffled all the art of the doctors. I stayed a few days at New York after your brother went, and there, by accident, I lighted on Dr. Warren's treatise of the yellow fever at Barbadoes, a book well known in London, and finding his description answer in most points to the Philadelphia sickness, I dispatched it immediately to Dr. Grœme, who was much assisted by it; and, after the arrival of the book, the distemper came to be well understood, so as to yield soon, in very violent cases, to a free use of sudorifics—Dr. Warren's specific for the cure. One particular symptom attended the fever in Philadelphia which I suppose never appeared in Barbadoes, and that is a suppression of urine from the first attack. This was the case of poor ——; and when the urine was suppressed, there is no instance of any one's recovery. At present, we enjoy good health, and a fine season. According to the best accounts I am able to get, the number of the dead since the 1st of June amonnts to no more than 250."

Some mention is made of the appearance of the fever in 1744, but the testimony to that point is not clear, and it may be doubted whether it returned until the 1747, when, according to a letter of Dr. Franklin's, it continued to prevail till arrested by the accession of frost. In 1760 a few cases of the disease appeared, but it was not epidemic. Two years later it made its appearance in a malignant form, preceded by very hot weather, and, as usual, was arrested by frost.

"We have no precise information," says Dr. La Roche, "as to the mortality resulting from the disease on the occasion before us. Mr. Willing estimates the loss in the vicinity of the wharf just named at upwards of sixty; but this estimate must fall far short of the number of those who fell victims to the disease, for the late Dr. Rush, who, in 1762, was studying medicine with Dr. Redman, states—in a note-book kept at the time, and of which he gives an extract in his account of a subsequent epidemic—that the disease spread like a plague, carrying off daily, for some time, upwards of twenty persons. When, with this in view, we bear in mind that the number of cases in daily attendance must, judging from the expressions of Dr. Rush, and the statement of Dr. Redman, have been considerable; that the disease proved highly malignant, and prevailed during more than three months, we may naturally infer that the mortality was very large—amounting, probably, to several hundred."

The histories of this epidemic left by those who witnessed its progress are brief and imperfect. Dr. Rush who was then a student of medicine, recorded some of its features in his note-book, and Dr. Redman has left a short description of it, from which there can be no doubt that the epidemic was yellow fever. Very different is the information attainable in regard to the visitations of 1793, '94 and '97. The disease, while devastating New York and Charleston, left Philadelphia unassailed for nearly thirty years. In the meantime, the city had grown to more than a hundred years old, and had acquired an able body of professional men. These have left behind them many valuable documents relative to the epidemic of 1793, which may be said to form an era in the history of yellow fever, since, as remarked by our author, on the observations made on that occasion, is in no small measure based the earliest knowledge obtained respecting the phenomena, character, origin, and mode of propagation of that disease. Dr. La Roche gives a dismal picture of the moral condition of the afflicted city gleaned from the histories of the epidemic, written by Rush, Volney, Deveze, Carey, Helmuth and Nassy.

"Many of those who remained being afraid to walk the streets, lest they might meet with individuals impregnated with the seeds of the pestilential infection, shut themselves up in their houses. Thousands, of all sexes and ages, resorted to the smoke of tobacco as a preventive against the contagion. Some chewed garlic, and wore it about their persons. Others, carrying their caution to a still greater length, provided themseves with new lancets, to be used on themselves or their families, in order to avoid thereby contamination from the instruments of physicians or bleeders. Many houses, as we are informed, were scarcely a moment in the day free from the smell of gunpowder, burned tobacco, nitre sprinkled with vinegar, etc. Churches were deserted, or wholly closed—as also the Exchange, City Library, and public offices—while the greater number of public papers were discontinued. Never were the rooms of Philadelphia oftener and more thoroughly purified, scoured, and whitewashed, than on this frightful occasion. Few went abroad without protecting themselves by the constant application to their olfactory nerves of handkerchiefs, or sponges, impregnated with vinegar or camphor. Some were found carrying pieces of tarred rope in their hands or pockets, or camphor bags tied around their necks. Many never walked on the footpath, but took to the middle of the street to avoid being infected in passing houses where death from the disease had occurred. Acquaintances and friends avoided each other in the street, and contented themselves with a cold nod. Shaking of hands fell into disuse, and cases of persons shrinking back with affright at even the offer of the hand are recorded. A craped hat, or any other token of mourning, was carefully shunned; and many persons valued themselves on the skill and address with which they gained the windward of those they met, and all uniformly and hastily shifted their course at the sight of a hearse. (*Carey.*) "The streets," says Dr. Rush, "everywhere discovered marks of the distress of the city. In walking for many hundred yards few persons were met, except such as were in quest of a physician or bleeder, or the men who buried the dead. The hearse alone kept up the remembrance of the noise of carriages or carts in the streets. Funeral processions were laid aside. A black man, leading or driving a hearse, with a corpse on a pair of chain-wheels, and now and then half a dozen relations or friends following at a distance from it, met the eye in most of the streets of the city, at every hour of the day; while the noise of the same wheels, passing slowly over the pavements, kept alive anguish and fear in the sick and well every hour of the night."

"It was impossible to find carriers of the dead, as a number of those who had at first performed that sad office were soon infected by the disease. It became necessary, in consequence, to resort to carriages for that purpose. The number of attendants at funerals was greatly diminished, and everybody retired at the approach of a hearse, and windows and doors were shut as they passed. The day, frequently, would not suffice to bury all the dead, because no assistance was to be had in digging the graves. Much of this was, therefore, done by night."

By a majority of the physicians the epidemic was ascribed to importation from the sickly ports of the West Indies. The College of Physicians gave a decision in approval of that doctrine; but they did not pretend to say from what part, or at what time, or in what manner it was introduced. "Public report had derived it from several islands, had chased it from ship to ship, and from shore to shore, and finally, conveyed it at different times into the city, alternately by dead and living bodies," and it was on such fluctuating reports of the people that the opinion of the College was based.

But the opinion was not universal among the physicians of Philadelphia. Dr. Redman, the President of the College, Dr. Foulke and Dr. Leib dissented from the report; Dr. Hutchinson, the inspector of sickly vessels, in an official communication, remarked that it did not appear to be an imported disease; and Dr. Rush united with these physicians in assigning a local origin to the disease.

"As regards the mode of propagation of the disease, a greater unanimity of sentiment existed at the outset of the epidemic, and for some time after. By the very large majority of the physicians and the public generally—even by those who advocated the opinion of local origin—it was thought to be transmitted by contact or intercourse with the infected; in other words, through means of contagion. But, even at that early period, physicians were found who rejected such a belief, and taught that the cause of the extension of the fever was to be sought in the diffusion, through the agency of the air, of morbid exhalations, themselves the offspring of organic decomposition. Gradually, the doctrine of non-contagion gained converts, until, finally, the physicians of Philadelphia became divided into two almost hostile parties. From this moment may be dated the origin of the interminable dispute about contagion and non-contagion, which has continued ever since to occupy the attention of the medical profession in this and other countries."

The yellow fever of the following year was less fatal in its character, and there were physicians in Philadelphia at the time who denied its existence. During the two succeeding years there were sporadic cases in the city, but the disease was not epidemic. It is interesting to remark, that in the first of these years the epidemic prevailed extensively in the city of New York, carrying off in a few weeks upwards of seven hundred, and also in the cities of Norfolk and Charleston; and yet these facts failed to open the eyes of a majority of medical men to the folly of looking to foreign ports for the origin of the pestilence. Dr. La Roche attributes the escape of Philadelphia from the epidemic, at that time, to the favorable character of the prevailing weather.

In 1796 came another epidemic yellow fever. The range of the thermometer during the warm season of that year, though not so high as in 1793, was above 80 deg. of Fahrenheit. The mortality was less than that of the year just mentioned, but, according to Dr. Rush, amounted to nearly one thousand, and according to Dr. Pascalis to thirteen hundred.

Dr. La Roche thus speaks of the epidemic of 1798:

EPIDEMICS OF 1798, 1799, 1802 AND 1803.

Severely as the city of Philadelphia had been visited in 1797, and especially in the memorable autumn of 1793, it pleased Provdence to put its inhabitants, in 1798, to a trial of perhaps still greater magnitude. The epidemic of that year, happily the last of the kind I shall have to record, was the most extensive, in point of malignity and mortality, of any that had yet occurred in this city, and may, in these respects, be placed on a parallel with even those by which portions of Spain, and our own city of New Orleans, have on several occasions been devastated. It afforded the last great manifestation of the epidemic constitution of the atmosphere—so far, at least, as this city is concerned—which had evidently prevailed during several years, and given rise to so much distress, and which, at the season under consideration, gave evidence of its existence and wide diffusion by the elicitment of the disease not only in Philadelphia, but also in Boston, New York, Portsmouth, (N. H.), Newport, (R. I.), New London, Wilmington, (D.), Charleston, in most of which it assumed a severe form. True it is, as we shall see, the disease broke out the next year, and has done so at subsequent periods; but on those occasions, it appeared in a less violent epidemic garb, or showed itself only sporadically.

The spring of 1798 assumed, at the outset, a promising aspect. There was plentiful rain, and vegetation commenced early. But a change took place about the middle of April, when a snow storm, succeeded by very cold weather, injured the sprouting leaves and blossoms. The intense heat of summer came on suddenly; the thermometer of Fahrenheit rising, on the 1st of May, as high as 84°. However, this spell was of short duration, and during that month and June, the weather, though warm, was variable, and at times attended with frost. In July, the heat became considerable, and continued so throughout, excepting towards the middle of the month, when the temperature suddenly lowered in consequence of a hail storm which occured at some distance from the city. Towards the close of the month, the heat again became extreme, the thermometer ranging from 88° to 92°, and rising as high at 96°. Throughout August and September, the weather was marked by high temperature. During the latter part of May, and in June, part of July, and throughout August and September, the weather was very dry; and, as a consequence of the drought, whole fields were burnt up by the sun, and the crops of hay seriously injured.

The mean temperature of the season was, for

| | Morning. | Mid-day. | | Morning. | Mid-day |
|---|---|---|---|---|---|
| June | 66 47 | 76.83 | September | 59.50 | 72.43 |
| July | 67.61 | 79.77 | October | 48.26 | 60.61 |
| August | 73.90 | 83.23 | November | 29.53 | 42.06 |

It is well to remark, that the mean temperature of the first three months of that series—the most important in reference to the developement or diffusion of the cause of yellow fever—was not lower at mid-day than 79 deg. 94 min., an elevation greater by several degrees than that observed in other yellow fever seasons, but below that of 1793, when, as we have seen, it rose to 81 deg, 37 min. During the season, the thermometer rose forty-one times at mid-day (3 o'clock) above 80 deg.*

The localities where the fever made its appearance and prevailed, were in no better condition, in point of cleanliness, than those that had been the theatre of its ravages at former periods. The sinks and sewers were, as heretofore, filled with putrefying materials, and emitted the same effluvia. Condie and Folwell, though far from being friendly to the doctrine of local origin, call attention nevertheless, to the impure condition of the city. They speak of many vacancies on the banks of the river, covered with a thick bed of miry filth; of the wharves becoming filled up with impure materials from the adjoining streets, and emitting at low water, a very offensive stench. They specially refer to Pegg's Run, with its wide and miry bottom, and with its banks unimproved, and rendered still more injurious by being made a receptacle for the offals from slaughter houses, tan yards, etc.; of the offensive condition of privies in thickly inhabited places; of the entire want of these in Water street, and the offensive smell issuing from them everywhere during moist, calm, and sultry weather—particularly towards the close of summer. The Academy of Medicine, in like manner, called attention to the subject, dwelling particularly on the "putrid exhalations of alleys, gutters, and docks, and of the stagnating waters in the neighborhood of the city."†

The disease began this year early in August. In the July preceding, and especially during the very hot weather that occurred in that month, cholera morbus prevailed to a considerable extent among children, and occasioned great mortality. Dysentery, also was rife; and towards the middle and close of the month, several cases of bilious fever of a highly malignant grade occurred in the western part of the city; and, in general, proved fatal. Everything, indeed—continuity of high temperature, dryness of weather, accumulation of sources of morbid exhalations, and the nature of the prevailing diseases, as well as the appearance, about the same time, of the fever in other parts of the Union, seemed to presage a sickly season; yet it was not until the 7th of August that the disease was officially announced by the Board of Health, on information communicated by the College of Physicians. Assum-

* Caldwell, Fever of 1805, p. 17. Agreeable to the observations of Cadwallader Evans, the mean temperature of June was 77 deg., of July 82 deg., and August 86 deg. 5 min., giving an average, for the period of 81 deg. 8 min.—(Eclec. Repert., vii, p. 428.

† Condie and Folwell, p. 50; Currie, p. 12; Rush, Med. Inq., iv. 48.

ing rapidly a fearful character of malignity, and spreading far and wide, it prevailed from that period through the rest of the summer, and during the whole of autumn. From the 1st of October the fever gradually abated, probably from a deficiency of subjects; for many families having, in consequence of great diminution in the mortality, returned to their homes, the disease broke out with renewed violence, and continued to prevail until checked by the advent of frost. On the 1st of November its total extinction was officially announced by the Board of Health. (*Currie*, p. 136).

As in most other epidemics, the principal seat of the disease, on this occasion, was the river side and the adjoining streets. It began about Spruce and Walnut streets, and wharves; but after a short time, and before the close of August, spread in nearly every part of the city. About the same time it appeared, as it had done before, in the district of Southwark, and the village of Kensington. On this occasion, and this only, the disease penetrated into the jail, situate at the corner of Sixth and Walnut streets,* and even into the Pennsylvania Hospital, in Pine, between Eighth and Ninth streets.†

The pathognomonic symtoms of the fever were the same this season as they had been on former occasions. The disease appeared, however, to possess a greater malignancy of character, and presented some peculiarities of phenomena, a notice of which will find a more appropriate place in another part of this volume.

The mortality in this memorable epidemic was larger—not in the aggregate, but in proportion to the number of individuals attacked, or those who continued exposed to the infection—than it had been during the autumn of 1793. The whole number of deaths reported to the Board of Health from the beginning of August to the close of the epidemic, was 3,645. But it is supposed that the fatal cases that occurred in the country would, if fully ascertained, swell the number to four thousand. Of these reported deaths, 514 occurred in the City or Fever Hospital, and 3,131 in the city and liberties.

From the following table it would appear that the disease attained—as was the case in 1797—its highest intensity in September:—

| | |
|---|---|
| August (from 8th) | 626 |
| September | 2,004 |
| October | 943 |
| From 1st to 5th November | 72‡ |

When we take into consideration the state of the population at the time of this epidemic, this mortality must, as that which had occurred five years before, appear very large. The whole number

* Currie, pp. 79, 144; Condie and Folwell, p. 75.
† Additional Facts, p. 33.
‡ Condie and Folwell, pp. 105, 6, 7, 8.

of inhabitants in the city and liberties in 1800 amounted, as we have seen, to something more than 70,000; but by the historians of the epidemic, the number, in 1798, was not estimated at more than 60,000.* Assuming the latter as the probable amount, it would follow that there died, during three months, 1 in 16.48 of the entire population. This proportion, which, under all circumstances, would appear large, must cease, to be so considered when we learn the state of public mind from an early period after the outset of the fever, and the result it produced almost immediately on the movement of the population. So early as the 7th of August, at the recommendation of the College of Physicians, the Board of Health suggested to the inhabitants of the infected district the necessity of removing into more healthy parts. The Academy of Medicine, in an official communication published on the 8th, recommended the same measure, as also that means should be taken to prevent the contaminated parts from being visited by the citizens; that all ships, and putrid articles of commerce, should be removed from the wharves and stores of the city; that the docks, wharves, yards, and cellars should be cleansed; that the gutters should be washed daily; that a sufficient number of physicians should be appointed to take care of such of the poor as might be affected with the fever; that the citizens should be advised to avoid all the usual exciting causes of fever—intemperance, fatigue excessive heat, the night air, and all violent and debilitating passions of the mind; and in every case of indisposition, slight in appearance, to apply immediately for medical however aid.†

These recommendations, though proper and advisable, called in a forcible manner, the attention of the public to the existing danger. Terror soon seized upon all classes. The alarm became general, and emigration was carried to an extent far beyond what had occurred in preceeding times of the calamity. The public offices and the banks were removed to the adjoining villages, and even physicians, few in number it is true, announced

---

* Condie and Folwell, p. 55.

Currie gives a different enumeration; from 1st of August to 3d of November, 3,446.

| | |
|---|---|
| August | 623 |
| September | 1,831 |
| October | 942 |
| November | 50 |
| | 3,446 |

He adds 60 after the 3d, making 3,506; and 300 in the country. (Currie, p. 129.)

Mease says 3,637:

| | |
|---|---|
| August | 626 |
| September | 2,004 |
| October | 943 |
| November | 64.—(p. 37.) |

† Condie and Folwell, pp. 47, 48.

their intention of leaving the city.* From Condie and Folwell, we learn that the number of the inhabitants who fled was variously estimated at from three-fourths to five-sixths of the population. They feel disposed to view the first as probably the correct estimate, and lay down at 40,000 the number who sought refuge in the purer air of the neighboring towns, or of the country (*Ib.* p. 55); leaving a comparatively small number exposed to the infection and to share the loss occasioned by it.

Some idea may be formed of the malignancy of the fever from the fact of the immense proportion of deaths to the number of persons attacked. The number of cases reported to the Board of Health amounted to 4,718, including 898 received at the City Hospital. Now, if we bear in mind the deaths reported fell but little short of 3,700 (3,645), we arrive at results of an almost unprecedented character. The proportion of deaths to the attacks was as 1 to about 1.27. That of the mortality in the City Hospital was as 1 to 1.75—the cases being 898, and the deaths 514; while in the city and liberties, where the number of cases amounted to 3,820, and the deaths to 3,131, we have a proportion of 1 in 1.23. These reports commenced only on the 18th, and the admissions at the hospital on the 7th. The deaths, however, till the 7th were 53. We may suppose that, from the commencement of the epidemic to the 18th, there may have occurred 150, which, when added to 4,718, gives us 4,868, or 1 death to 1.34.

The mortality in the early part of the epidemic was less severe than it was subsequently; for, while the proportion of deaths to cases throughout was as one 1 to 1.297, that from the 9th August to the 19th September was as 1 to 1.34—the cases reported being 1,937 and the deaths 1,424. In the hospital, the cases amounted to 535, and the deaths to 276—giving a proportion of 1 in 1.2, or thereabout, instead of 1 in 1.75.†

WHOLE NUMBER OF CASES REPORTED TO THE BOARD OF HEALTH, AND THE RESULT THEREOF.

| | Cases Reported. | Deaths. | Proportion. |
|---|---|---|---|
| August | 827 | 626 | 1 in 1.32 |
| Adding | 150 | | 1 in 1.56 |
| | 977 | | |
| September | 2,969 | 2,004 | 1 in 1.43 |
| October and November (from 1st to 5th no admissions) | 922 | 1,015 | 1 in 0.908 |
| | 4,718 | 3,645 | 1 in 1.297 |
| Adding the 150 | 150 | | |
| | 4,868 | | 1 in 1.34 |

* Condie and Folwell, p. 57.

† Currie, pp. 84, 85; Condie and Folwell, Appendix, p. xiv.

CITY HOSPITAL.

| | Cases Reported. | Deaths | Proportion. |
|---|---|---|---|
| August | 222 | 112 | 1 in 1.2 |
| September | 483 | 276 | 1 in 1.75 |
| October | 193 | 126 | 1 in 1.53 |
| | 898 | 514 | 1 in 1.75 |

The great mortality of this season, in proportion to the remaining population, finds a ready explanation, doubtless, in the greater diffusion of the poison, but, also, in the fact that all, or nearly all, the inhabitants in easy and comfortable circumstances had fled from the infected city, and that the subjects on whom the disease bore were mostly to be found among the lower classes, who, residing in confined, ill-ventilated, and filthy courts and alleys, and unable to seek the benefit of a purer atmosphere, were more exposed to the influence of the morbific cause. The emigration in 1793 did not exceed, as we have seen, one-third of the inhabitants. Hence, among those who remained, a larger portion were of the better classes, who are less prone than the others to be attacked with the disease, and, when so attacked, have it in a lighter form; whereas, in 1798, few of that privileged class felt disposed to confront the danger. As regards the greater mortality in proportion to the number attacked, it was due solely to the greater malignancy of the disease, which, owing to this circumstance, combined with the unfavorable conditions of the classes attacked—arising from the causes just mentioned, as well as bad nursing, etc.,—baffled all the efforts of art, and occasioned nearly as many victims as it did sufferers. Dr. Rush, in taking notice of this disproportion of deaths to the number attacked, attributes it to another cause. It "was owing," he says, "to the liberal and general use of the lancet in 1793, and to the publications in 1797 having excited general fears and prejudices against it in 1798. Such," he continued, "was the influence of these publications, that many persons who had recovered from this fever in the two former years, by the use of depleting remedies, deserted the physicians who had prescribed for them, and put themselves under the care of physicians of opposite modes of practice. Most of them died."

The sufferings to which the poor and destitute were exposed during this public calamity, from the apprehension entertained of contagion, the wide diffusion and fatal tendency of the disease attained, and the panic that had seized upon every class of the community, an extent unparalleled in the annals of this country. Trade was completely paralyzed, intercourse was almost suspended, the work ing people were without employment; and while their dearest affections were torn asunder by the constant inroads of the disease, starvation threatened to fill the bitter cup of their sufferings. The public prints of the day, as also the work of Condie and Folwell

—the faithful chroniclers of the epidemic—contain details calculated to make every human heart bleed. It is gratifying to discover in the history of the time, that those who fled from the city were more hospitably and humanely received and treated in places where they had sought shelter than had been the case formerly.* Experience had already shown, that no fear of contagious contamination was to be apprehended from the effect of emigration.

By some, the early cases were attributed to a contagious virus, which had remained dormant from the preceding autumn in the beds and bedding of individuals who had died of the disease. The greater number attributed, as usual, the fever to importation from the West Indies, and vessels were confidently pointed out—the ship Deborah, Capt. Yard, from Jeremie; the schooner Aurora, from Cape Nicholas Mole; the Ariel, from the same; the brig Mary, from Kingston, Jamaica, etc. Faithful to the views he had heretofore entertained and expressed relative to the origin of the fever, Dr. Rush rejected the idea of importation, and referred the calamity to the operation of local sources of infection. Many other physicians—and the Academy of Medicine as a body—adopted the same opinion, which was destined to become soon that of the large majority. While such was the sentiment of these in relation to the origin of the fever, the greater number among them, and all the advocates of importation, adhered to the doctrine of contagion, and affirmed that, however the disease might originate, it was communicated from the sick to the well. But the events of the preceding year had induced some to modify their views on the subject, to limit the possibility of contagion within very narrow bounds, or even to deny it altogether. Those of the present sickly season made new converts. Not a few traitors were now to be found in the camp; and it is from this period we must date the revolution in Dr. Rush's mind relative to the subject, which he frankly avowed a few years after."

The fever reappeared the year following, but in a mitigated form and with less disposition to spread. It broke out earlier in the season than it had done the previous year, and spread slowly during the course of July, declining towards the beginning of August, and soon after almost disappearing for a time, but to revive and spread more rapidly and extensively until arrested by a thick frost in October. The mortality was comparatively small; Dr. Mease gives it at a little over 1,000, but this grew in part out of the general flight of the citizens. As on former occasions, the College of Physicians assigned to the epidemic a foreign origin,

---

* Condie and Folwell, p. 55.

but by the Academy of Medicine, of which Dr. Rush was a leading member, it was held to be generated in the city by domestic morbific exhalations.

During the summer and autumn of the two years succeeding the fever did not appear in Philadelphia except in sporadic cases; but while this city was comparatively exempt from the pestilence, New York, Providence, (R.I.), Norfolk, Baltimore, and Charleston suffered by its visitatious.

Very different was the result in the year following, 1802, when the yellow fever once more made its appearance among us in the epidemic form. But, on this, as on other occasions, it reigned in a less general manner, and proved a source of much less distress and mortality than it had at several former epochs. I cannot find that anything in the character of the weather or temperature of the spring or early summer months, could have led any one to anticipate a greater developement of the disease than had occurred in 1800 and 1801. The month of March, as we learn from Dr. Rush, was wet, cold, and stormy, with the exception of a few pleasant days. Frost was common in April and in May. The weather was so cool as to make fires agreeable to the last, the wind blowing chiefly during the time from the northeast; while in the month of June, the weather was cold, rainy, and hot in succession; and, during the first two weeks of July, there fell a considerable quantity of rain. As regards the degree of temperature during the summer, June presented, at 3 P. M., a mean of 75.7; July a mean of 78; and August a mean of 78; the result for the three months being 77.2.*

The fever was preceded by the scarlatina anginosa, which, together with the cynanche trachealis, was the principal disease during the winter months. It reigned almost paramount during May and June, and extended into the succeeding month.

While Philadelphia was thus once more visited by the malignant fever, other cities of the Union suffered in an equal and even greater degree. It prevailed extensively in Baltimore, and especially at Wilmington, Del., where its progress, from the outset to the close, was marked by the utmost severity. It also prevailed in Charleston, while sporadic cases of the disease occurred in New York, Boston, and Portsmouth, N. H. Here it made its appearance, in isolated cases, on or about the 4th of July; the disease, in these instances, being sufficiently well characterized to be recognized as such by Drs. Currie and Cathrall, (pp. 6. 7). Its epidemic career did not commence, however, before the 15th of July, when it broke out near the corner of Front and Vine streets. On the

* Cadwallader Evans, Eclectic Rep., vii. p. 428.

2d of August, it extended to other parts of the city, particularly Front and Water streets, near the drawbridge, as well as to Chesnut street, near the wharf. The disease continued to prevail in different parts of the city during the months of August and September. The number of the cases increased after a white frost. The fever declined after a black frost which occurred on the 28th of October, and ceased entirely early in November.

As may very readily be imagined, the epidemic diffusion of the yellow fever in Philadelphia was a source of considerable alarm to the inhabitants, who had passed through many severe ordeals of the kind. But this alarm, great as it might have been, was much increased by the course pursued by the Board of Health. On the 4th of August, it publicly announced that the disease had made considerable progress, and that the mortality had been very alarming since its reappearance; though every part of the city and liberties, as far as they could learn, excepting the neighborhoods adjacent to the place where the fever originally appeared, remained remarkably healthy, and entirely exempt from any disease marked with malignant or dangerous symptoms. Dreading; as was stated, the renewal of the melancholy scenes of former years, and finding it would be impracticable, with their limited powers, to prevent intercourse with the infected, they declared the fever to be contagious, and recommended to the citizens to withdraw into the country. The advice, says Dr. Rush, was followed with uncommon degrees of terror and precipitation; and the almost entire population poured into the country, where they remained until publicly invited to return by the Board of Health on the 5th of October.

The mortality from the fever amounted, according to one authority,* to 835, and was distributed as follows: August, 262; September, 284; and October, 289. By Drs. Currie and Cathrall, the number is differently estimated. According to these writers, the mortality from all diseases, from July to October, inclusive, amounted to 1,108; *i. e.*, July, 261; August, 262; September, 284; October, 301 (p. 17). The number reported as affected, during the epidemic, was 598; of which number 307 died. During the same period, there died of other diseases 505 persons; making, altogether, 1,069. (*Ib.* p. 17). In the hospital, 110 patients were received, and of these 58 died. Admitting these statements to be correct, it follows that the proportion of deaths to cases was as 1 to 1.68. In the hospital, it was 1 to 2, or fifty per cent. The greatest mortality, in proportion to the number of sick, occurred between the 15th and the 25th of October.

The succeding year was another epidemic, but less diffused and less fatal than many of its predecessors. It was preceded and

* Mease, op. cit., p. 38.

accompanied by remittent and intermittent complaints. About the latter part of July cases appeared, but a favorable change of weather taking place, the fever was stopped and the city remained free from it until the close of the month of August when it broke out afresh with more severity than before. It spread extensively in September, but early in October it had greatly declined, and on the 20th of that month the city received the joyful announcement of of the extinction of the disease. The proportional mortality was small, being, according to Dr. Rush, about five in a hundred in the practice of most physicians, and the absolute mortality was less than 200. By this time the doctrine of the local origin and non-contagious nature of the fever made considerable progress in the profession, though the public continued to adhere as pertinaciously as ever to the old and contrary opinion.

In 1805, 1820, and 1853 the city again suffered incursions of the epidemic, but each in a form less malignant than the preceding.

Such is an outline of Dr. La Roche's history of yellow fever in Philadelphia, and with it we must close, for the present, our notice of his work. In our next number we shall present an analysis of other portions of it.

